# Bryostatin Modulates Latent HIV-1 Infection via PKC and AMPK Signaling but Inhibits Acute Infection in a Receptor Independent Manner

**DOI:** 10.1371/journal.pone.0011160

**Published:** 2010-06-16

**Authors:** Rajeev Mehla, Shalmali Bivalkar-Mehla, Ruonan Zhang, Indhira Handy, Helmut Albrecht, Shailendra Giri, Prakash Nagarkatti, Mitzi Nagarkatti, Ashok Chauhan

**Affiliations:** 1 Department of Pathology, Microbiology and Immunology, University of South Carolina School of Medicine, Columbia, South Carolina, United States of America; 2 Department of Medicine, University of South Carolina School of Medicine, Columbia, South Carolina, United States of America; 3 Department of Experimental Pathology, Mayo Clinic, Rochester, Minnesota, United States of America; Tsinghua University, China

## Abstract

HIV's ability to establish long-lived latent infection is mainly due to transcriptional silencing in resting memory T lymphocytes and other non dividing cells including monocytes. Despite an undetectable viral load in patients treated with potent antiretrovirals, current therapy is unable to purge the virus from these latent reservoirs. In order to broaden the inhibitory range and effectiveness of current antiretrovirals, the potential of bryostatin was investigated as an HIV inhibitor and latent activator. Bryostatin revealed antiviral activity against R5- and X4-tropic viruses in receptor independent and partly via transient decrease in CD4/CXCR4 expression. Further, bryostatin at low nanomolar concentrations robustly reactivated latent viral infection in monocytic and lymphocytic cells via activation of Protein Kinase C (PKC) -α and -δ, because PKC inhibitors rottlerin and GF109203X abrogated the bryostatin effect. Bryostatin specifically modulated novel PKC (nPKC) involving stress induced AMP Kinase (AMPK) inasmuch as an inhibitor of AMPK, compound C partially ablated the viral reactivation effect. Above all, bryostatin was non-toxic in vitro and was unable to provoke T-cell activation. The dual role of bryostatin on HIV life cycle may be a beneficial adjunct to the treatment of HIV especially by purging latent virus from different cellular reservoirs such as brain and lymphoid organs.

## Introduction

Introduction of highly active antiretroviral treatment (HAART) is able to successfully control HIV viremia in most AIDS patients and has remarkably reduced the incidence of HIV-associated neurological complications [1]. While an undetectable viral load is achieved in most HAART treated patients; latent viral reservoirs continue to harbor HIV proviral DNA permanently in resting memory CD4^+^ T cells [2]–[7]. There are several mechanisms proposed for HIV latency including cellular factors acting as restriction factors, RNA interference, integration of the proviral DNA in transcriptionally dormant site that may be derived from methylation status, Tat activated elongation factor (P-TEFb), histone modifications or unavailability of cellular transcription factors like NF-κB that act as co-activators of the HIV LTR [8]. HIV post integration latency is mainly due to transcriptional silencing that involves chromatin reorganization.

Current antiretroviral therapy lacks a component capable of reactivating latent viral infection. This latent viral reactivation component is essential along with HAART to purge the virus from compartmentalized latent viral reservoirs. Latent HIV responds to T-cell activation signals [9]–[15]. T-cell activation strategies include treatment with proinflammatory cytokines such as IL-6, TNF-α, IL-2, and in monocyte/macrophages IFN-γ. However, these combinations lead to T-cell depletion and rebound in viral load when HAART is withdrawn. Moreover, IL-7 also reactivates latent HIV infection; however, it contributes to maintain latent reservoir in patients with low CD4^+^ cell counts [7], [16]–[19]. Overall, the relevance of such immune activation strategies is not considered promising and T-cell and TCR stimulation was found to be associated with significant toxicity. New evidence has shown the existence of other latent reservoirs such as CD14^+^CD16^+^ monocyte phenotype and hematopoietic stem cells in the bone marrow [20]–[23]. Among HIV patients, monocytic cells are known to undergo latent infection and are refractory to HIV inhibitors. Macrophages have also been proposed to harbor latent virus. As a proof of principle, in SIV infected macaques, CD34^+^ CD4^+^ monocyte progenitor cells were shown to be infected early in infection and harbor latent infection [24], similar to HIV infected patients [25]. Above all, several recent studies have revealed that patients on HAART support the existence of other stable viral reservoirs in addition to latently infected resting memory CD4^+^ T cells [26]–[29].

Histone deacetylases (HDAC) promote latency by regulating genome structure and transcriptional activity. HDAC inhibitors (Trichostatin A [TSA], valproic acid [VPA], sodium butyrate, suberoylanilide hydroxamic acid [SAHA]) and the PKC activators (VPA, PMA and prostratin) have been investigated for their broad spectrum latent viral reactivation in T-lymphocytes and monocyte/macrophages. A family of serine/threonine kinase isoenzymes ‘PKCs’ is activated normally by external stimuli on the plasma membrane receptors coupled to phospholipase C. Once activated, PKCs exert a variety of effects by phosphorylating their downstream substrates. Depending on cell type, these include receptor desensitization, cell proliferation and apoptosis. DNA topoisomerase II is one of the substrates for PKC and inhibition of PKCs lead to reduced levels of phosphorylated DNA topoisomerase II, thus leading to inhibition of HIV infection [30]. PKC signaling reactivates latent HIV infection involving several activated factors such as NF-κB, NF-AT and AP1. The most attractive PKC agonists are non mutagenic, non tumorigenic prostratin and SAHA, which reactivate latent HIV in lymphoid and myeloid cells despite minimal immune activation and perturbation of cell cycle progression. Although introduction of valproic acid and SAHA (Vorinostat) were envisioned to flush out the latent virus from these reservoirs within few years, valproic acid with HAART failed to deplete latent HIV reservoir [31]–[34]. Despite HDAC inhibitors advantages and clinical approval for cancer and epilepsy, they inhibit general gene transcription and in particular alter the gene expression in transformed cells [35], [36]. However, SAHA did not induce cell surface activation markers on bystander T-cells [37]. Although SAHA was more potent than valproic acid, the induced side effects such as diarrhea, fatigue, nausea, anorexia and dehydration will likely render it non-suitable for long term use [38], [39].

Bryostatin-1 which is a member of the group of bryostatins, is a macrocyclic lactone, isolated from a marine bryozoan *Bugula neritina* [40] produced by its endosymbiont γ-proteobacterial *Endobugula sertula* [41]. Bryostatins are known for their anti-neoplastic activity and PKC activation. Bryostatin also causes dephosphorylation of CDK2 which inhibits RNA polymerase-II phosphorylation [42], thus impairing HIV Tat function [43]. Bryostatin-5 has been reported to block stromal cell derived factor-1 (SDF-1), a natural ligand of CXCR4 receptors [9].

We therefore, sought to investigate the potential of bryostatin-1 in viral reactivation using latently HIV-infected monocytic as well as lymphocytic infection models. We also investigated the anti-HIV activity of bryostatin in acute infection models of X4- and R5-tropic viruses on Jurkat and Magi cells as well as the mechanism of bryostatin mediated regulation of latent HIV-infection.

## Results

### Bryostatin Inhibits HIV-1 Infection in Lymphocytes

**Figure 1:**
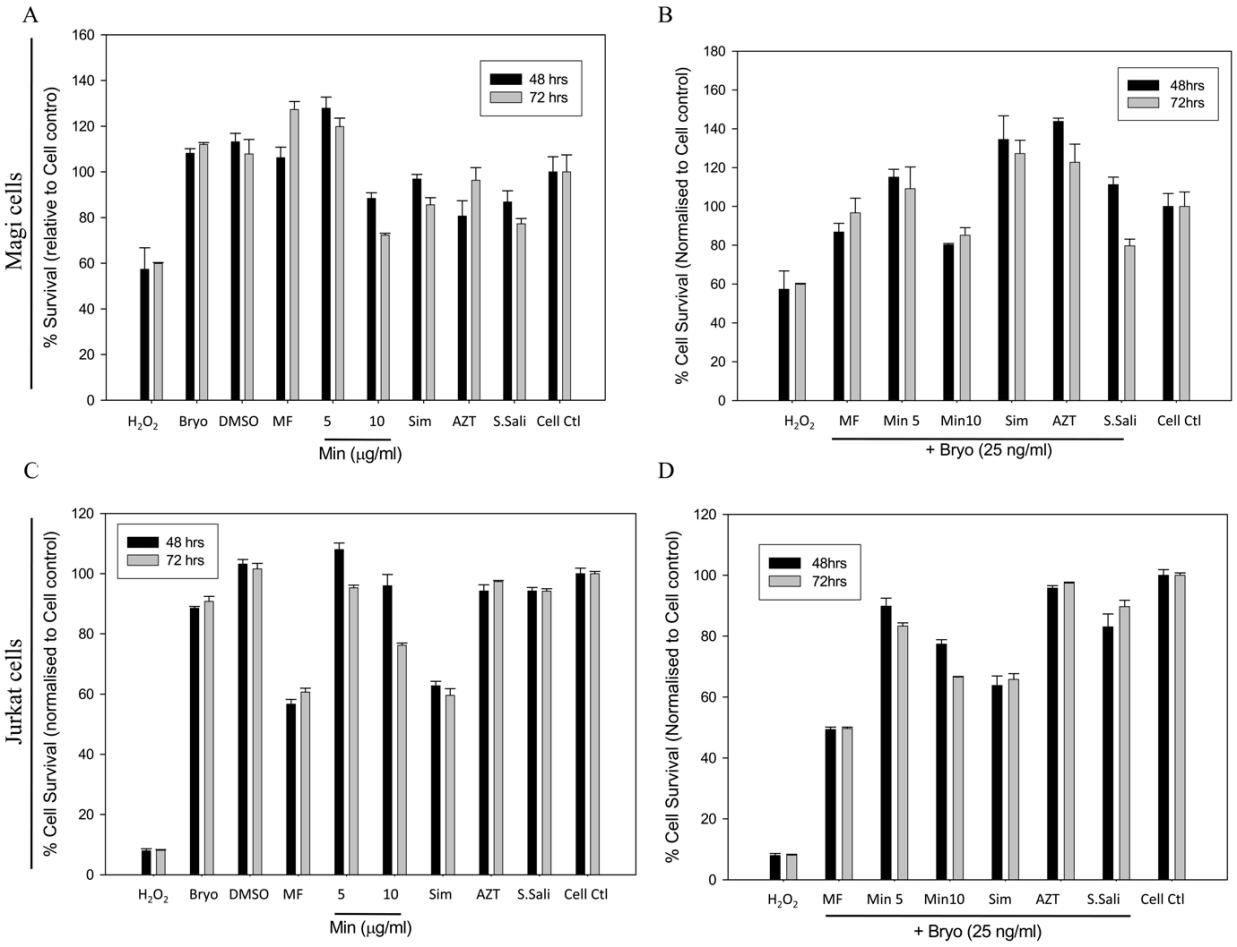
Bryostatin is non-toxic at its active concentration either alone or in combination with other anti-HIV compounds. Magi and Jurkat cells were treated with various therapeutic drugs (MF-Metformin (1 mM), Min-Minocycline (5 µg/ml and 10 µg/ml), Sim-Simvastatin (2.5 µM), and AZT (100 µM), S.Sali-Sodium salicylate (1 mM) either alone or in combination with bryostatin for 48 and 72 h and cell survival was determined using WST8/CCK8 cytotoxicity assay. (**A**) Survival of Magi cells after treatment with bryostatin and other drugs, (**B**) bryostatin in combination with other drugs. (**C**) Jurkat cell survival after treatment with bryostatin (25 ng/ml) and other drugs, (**D**) bryostatin in combination with other drugs. Each treatment was done in triplicate. (n = 2).

**Figure 2:**
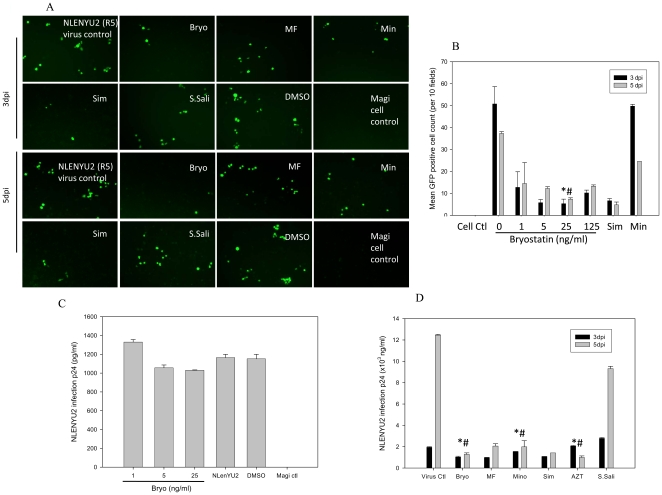
Bryostatin ablates R5-tropic HIV infection. Magi cells (CD4^+^/CCR5^+^) were infected with 100 ng/ml p24 equivalent HIV recombinant NLENYU2 virus expressing YFP. (**A**) Productive viral infection was monitored semi-quantitatively on 3rd and 5th dpi. (**B**) GFP-positive cells per 10 fields were counted in HIV-infected cultures and plotted as mean. (**C**) Dose dependent anti-HIV effect of bryostatin was monitored by p24 assay. (**D**) p24 levels in the culture supernatants after treatment with bryostatin (25 ng/ml) and other compounds on 3rd and 5th day post infection are shown. Sodium salicylate (1 mM) was used as a negative control in each set of experiments. Degree of significance for bryostatin treatment was relative to virus control, * p<0.05, # p<0.001. (n = 4).

**Figure 3:**
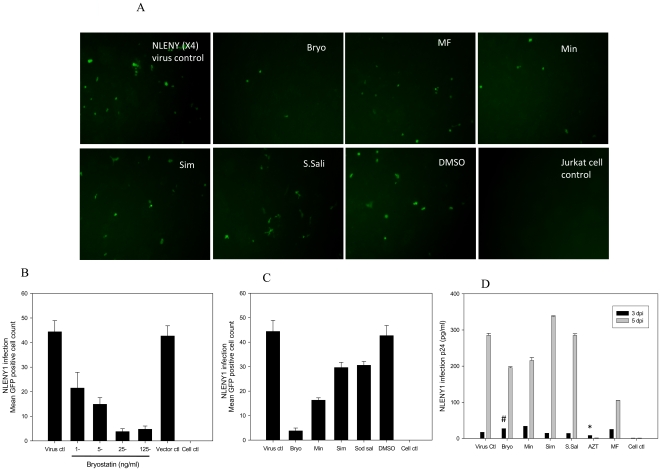
Bryostatin ablates X4-tropic HIV infection. Jurkat cells were pretreated either with bryostatin or other drugs, infected with 50 ng/ml p24 equivalent recombinant NLENY HIV-1 expressing YFP followed by continuation in respective treatments. (**A**) YFP expression levels were monitored semi-quantitatively (data shown in representative pictures on 5th dpi). (**B**) Bryostatin treatment ablated HIV infection as demonstrated by decrease in YFP-positive Jurkat cells on 5th dpi. (**C**) Productive HIV replication was monitored by YFP-positive cells on 5th dpi and (**D**) validated by p24 levels in infected culture supernatants on 3rd and 5th dpi. Results were statistically significant (#p<0.005, * p<0.0001) compared to HIV-1 NLENY virus control. (n = 3).

**Figure 4:**
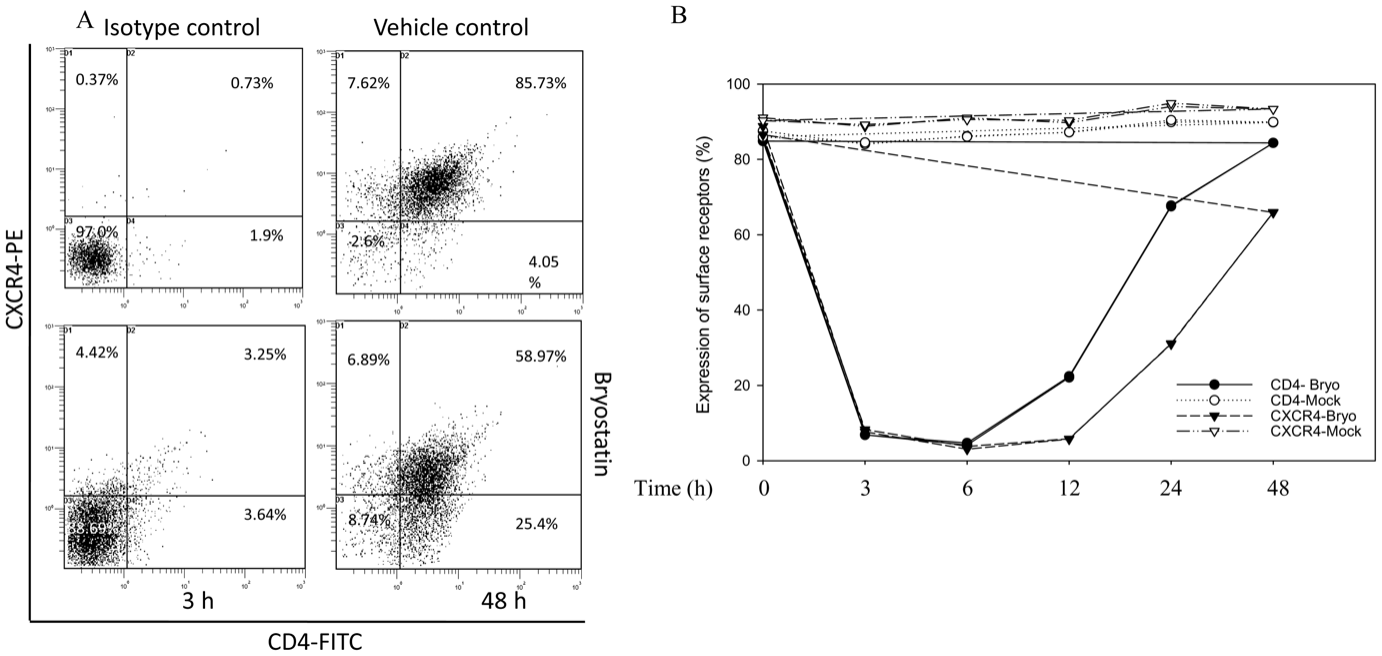
Bryostatin down-regulates CD4 and CXCR4 receptor expression on Jurkat cells. (**A**) Jurkat cells were treated either with bryostatin (25 nM) or left untreated for 0 to 48 h followed by immunostaining with fluorescently labeled anti-CD4 and anti-CXCR4 antibodies and monitored by flowcytometry. Representative dot plots at 3 h and 48 h of bryostatin treatment, isotype control and vehicle control (DMSO). (**B**) Time course of expression profile of CD4^+^/CXCR4^+^ receptors on Jurkat cells upon treatment with bryostatin (n = 2).

**Figure 5:**
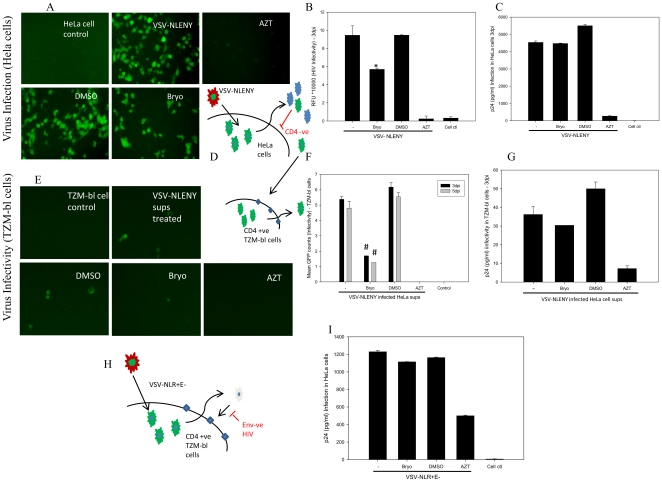
Bryostatin inhibits HIV-1 independent of viral receptors. (**A** and **B**) Hela cells were pretreated with bryostatin (27 nM), vehicle, and AZT as positive control and thereafter infected with VSV pseudotyped-NLENY1 HIV. Productive HIV replication was monitored using YFP expression after 3dpi. Results were significant at p<0.05 (**C**) Virus production was further confirmed by p24 levels in infected culture supernatants by quantitative ELISA. (**D**) Schematic representation of the experimental design of virus infection in HeLa cells and infectivity assay in TZM-bl cells is shown. (**E** and **F**) Virus infectivity in the bryostatin treated HIV-infected culture supernatants was monitored by infecting TZM-bl cells either with YFP expression as a marker or (**G**) viral p24 production after 3dpi. (**H** and **I**) Schematic representation of single round infection experiment, bryostatin mediated HIV inhibition was monitored by p24 assay in a single round infection on Hela cells using VSV pseudotyped HIV-NLR+E- (*p<0.05, #p<0.005) (n = 2).

Because bryostatin has been shown to down regulate CD3 and CD4 receptors on T-cells [46], we investigated whether bryostatin could confer anti-HIV activity against X4- and R5-tropic strains. Initially, bryostatin was investigated for any adverse effects on Jurkat and Magi cells (CD4^+^/CCR5^+^) in a dose response manner. Bryostatin failed to invoke cytotoxic response even at high doses as shown in cell survival assay (**Fig. 1**). In order to study the effect of bryostatin in acute infection and address whether bryostatin can enhance or suppress HIV replication, before going directly to latent viral reactivation studies we performed HIV infection studies on Jurkat and Magi cells in the presence of variable concentrations of bryostatin. Interestingly, bryostatin treatment of acutely HIV-infected cultures demonstrated mild anti-HIV activity in a dose dependent manner against both R5- and X4-tropic viruses compared to the untreated infected control on 3^rd^ and 5^th^ day post infection (**Figs. 2** **,** **3**), albeit, there was no complete suppression of HIV infection compared to AZT which was used as a positive control (**Fig. 2A**). There was an upregulation of viral p24 in bryostatin treated HIV-infected cultures similar to untreated infected cultures on the 3^rd^ day post infection (pi), however, on the 5^th^ day pi, p24 levels declined markedly compared to untreated virus control (**Fig. 3D**). Simvastatin was used as a control and failed to reduce p24 expression in acute HIV infected cultures, however revealed some anti HIV activity when monitored by GFP expression probably due to less sensitivity of GFP. In our earlier studies, Simvastatin has manifested week anti HIV activity [45]. Overall, bryostatin curtailed active viral replication in Jurkat (X4) and Magi cells (R5) as revealed by percent decrease in GFP positive cells (**Fig. 2** **,** **3**) which was validated by p24 antigen in the infected culture supernatants. To dissect the mechanism of HIV inhibition by bryostatin, we profiled the expression levels of CD4- and CXCR4-receptors on Jurkat cells after bryostatin treatment. Interestingly, bryostatin treatment at 25 ng/ml concentration down-regulated the expression profile of both CD4- and CXCR4-receptors when monitored by flowcytometry (**Fig. 4A**). Following time course treatment with bryostatin, both the receptors were found to be transiently down-regulated and returned to baseline levels within 48 h (**Fig. 4B**), consistent with earlier findings [46], [47]. Thus, these observations suggested the occurrence of another mechanism of bryostatin mediated HIV inhibition. In order to investigate whether bryostatin mediated HIV inhibition is independent of cellular receptors, we employed vesicular stomatits virus envelope (VSV-G) to pseudotype HIV NLENY1 (VSV-HIV) virus, wherein VSV-HIV (GFP expressing virus) enters the cells independent of CD4, CXCR4 and CCR5 receptors. Bryostatin treatment moderately inhibited VSV-HIV infection independent of receptors in Hela cells as revealed by decrease in virus productivity and virus infectivity in TZM-bl cells (**Fig. 5A–G**). Bryostatin showed similar results on Hela cells in a single round infection against envelope deficient HIV-1 NLR+E- strain pseudotyped with VSV-G envelope (**Fig. 5H, I**), albeit revealed HIV inhibitory activity of bryostatin in low nanomolar concentrations such as 25 ng/ml (27.6 nM), suggesting the occurrence of additional effects on HIV replication. All together, these data suggested that bryostatin can abrogate the HIV infection in a receptor independent manner.

### Bryostatin modulates HIV Latency via PKC Signaling

**Figure 6:**
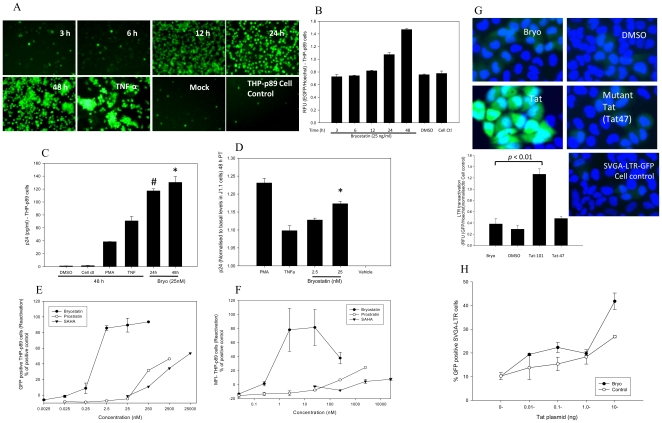
Bryostatin reactivates latent HIV infection in monocytic cells. (**A**) THP-p89 cells were treated with bryostatin (25 ng/ml) for different durations and fluorescence was quantified by flowcytometry. (**B**) GFP expression was directly proportional to the level of activation in these cells as visualized by fluorescence microscopy. Quantification of GFP fluorescence in bryostatin-treated latently-infected THP-p89 cells was done by flowcytometry. (**C**) Bryostatin primed viral reactivation at 24 and 48 h, was followed by release of viral p24 in culture supernatants using ELISA. (**D**) Bryostatin induced viral reactivation was further confirmed on another latently HIV-infected lymphocytic cell model (J1.1), 48 h post treatment using viral p24 as a marker. (**E** and **F**) Comparative dose response of bryostatin, prostratin and SAHA, for viral reactivation in THP-p89 cells. Bryostatin at a 1000 fold lower concentration, EC50<0.25 nM reactivated latent HIV in THP-p89 cells more potently than SAHA and prostratin (# p<0.001, * p<0.005). (**G** and **H**) Bryostatin in absence of Tat failed to activate HIV promoter. SVGA-LTR-GFP cells were either treated with bryostatin, vehicle control or transfected with wild type Tat-expression vector (positive control) in parallel with control mutant-Tat expression vector (negative control). The LTR transactivation was measured after 48 h by GFP fluorescence. Nuclei were stained with Hoechst 33342. (**G**) GFP expression visualized by fluorescence microscopy; pictures and bar graph are showing level of fluorescence. (**H**) Dose response for Tat-expression vector with or without bryostatin (25 ng/ml) in SVGA-LTR-GFP cells (n = 3).

HIV-latent reservoirs are established in T-cells and monocyte/macrophages in the body and maintain the infection for the entire life of the individual. Bryostatin is a known modulator of PKC isoforms [48] which are known to have the potential to reactivate latent HIV infection. Hence, we sought to investigate whether bryostatin can reactivate latent HIV-infection in monocytic cells. THP-p89 cells were established by infection with recombinant HIV-p89-GFP virus (dual tropic) and latent cells were negatively selected by FACS and verified by treatment with either TNF-α (10 ng/ml) or TSA (100–300 nM). Following treatment with bryostatin, latently HIV-infected THP-P89 cells were profoundly reactivated with evidence of GFP expression within 24 h compared to negative control (**Fig. 6A, B**). In parallel, TNF-α was used as a positive control and DMSO (vehicle) as negative control. Bryostatin mediated GFP expression in reactivated THP-p89 cells was confirmed by an authentic viral replication marker p24 in the supernatants at 24 and 48 h (**Fig. 6C**). Bryostatin reactivated latent HIV-1 infection in monocytic cells more robustly than PMA and TNF-α (**Fig. 6C**). To corroborate the reactivation of latently HIV-infected monocytic cells, latently HIV-infected lymphocytic J1.1 cells were incorporated in the experimental design. Given that HIV undergoes latent infection phase in memory T-cells, lymphocytic J1.1 is an ideal in-vitro cell model to study regulation of HIV. Bryostatin, similar to THP-p89 cells provoked viral reactivation in latently HIV-infected J1.1 T cells (**Fig. 6D**). In parallel with bryostatin, PMA and TNF-α were used as positive controls in viral reactivation while DMSO (vehicle) was the negative control. Again, bryostatin showed higher reactivation than TNF-α but slightly lower than PMA. Recently, prostratin and SAHA were shown to be potential candidates for reactivation of latent HIV in clinical settings [37], [49], [50]. Thus, we sought to investigate the latent viral reactivation strength of bryostatin in comparison to prostratin and SAHA. Remarkably, bryostatin reactivated latent viral infection 1000-fold more potently than prostratin (phorbol ester) and SAHA (**Fig. 6E, F**). Further, we investigated whether latent HIV-reactivation by bryostatin was a direct effect on LTR-transactivation in the absence of Tat. We have established a Tat-regulated GFP-reporter system in SVGA cells and this was used in our experiments. Following treatment of SVGA-LTR-GFP reporter cells [14], bryostatin alone failed to transactivate HIV LTR compared to Tat-expression vector that was used as a positive control (**Fig. 6F**). Furthermore, to investigate any synergism between Tat and bryostatin, we transfected SVGA-LTR-GFP cells with a suboptimal concentration of a Tat expression vector either alone or in combination with bryostatin. Following transfection with wild type Tat or mutant Tat expression vector, we monitored HIV LTR activity by quantifying the number of GFP-positive cells using flowcytometry. Although, bryostatin in combination with Tat revealed a slight increase in GFP (**Fig. 6G and H**), this was statistically not significant (**Fig. 6G**). Hence, we inferred that bryostatin induced HIV-reactivation does not involve direct transactivation of HIV-LTR either alone or in combination with Tat.

### Bryostatin Mediates Reactivation of Latent HIV via Classical and Novel PKCs

**Figure 7:**
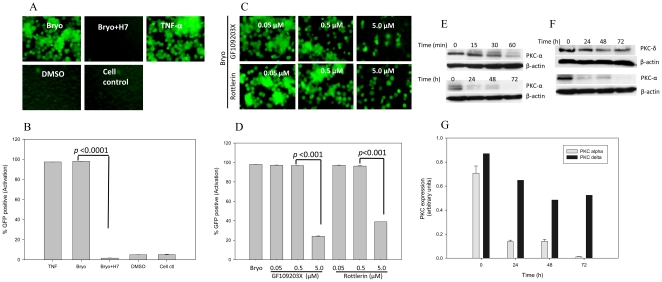
Bryostatin reactivates latent HIV infection via activation of classical and novel PKCs. (**A** and **B**) THP-p89 cells were pretreated with bryostatin either alone or with broad spectrum PKC inhibitor H7 dihydrochloride or (**C** and **D**) different concentrations of classical PKC inhibitor GF109203X and novel PKC inhibitor Rottlerin as indicated and monitored for GFP fluorescence by flowcytometry. (**E**) Total PKCα activation at different time points post-treatment with bryostatin. (**F**) PKC-δ and PKC-α in replicate experiment was degraded in a time dependent manner within 72 h of treatment with bryostatin. (**G**) Quantitative profile of PKC-α and δ after normalization with beta actin.

Bryostatin is a known potent activator of PKC that is active at low nanomolar concentrations. In addition, PKCs have also been shown to activate HIV transcription via NF-κB [51]. We sought to investigate whether bryostatin could reactivate latent HIV infection via modulating PKCs and identify which specific PKC isoform is involved in the activation loop. To dissect the HIV reactivation pathway, we inducted PKCs inhibitors along with bryostatin on latently HIV infected THP-p89 cells. Pretreatment with a broad spectrum PKC inhibitor, H7 dihydrochloride, completely annihilated bryostatin induced reactivation of latent HIV (**Fig. 7A, B**). Further, pretreatment with classical PKC (PKC-α, β1, β2, γ) inhibitor GF109203X at less than 0.5 µM concentration, followed by treatment with bryostatin, was not able to abrogate the effect of bryostatin on THP-p89 cells. However, higher concentrations of GF109203X (5 µM) inhibits other PKC isoforms non-specifically and therefore, attenuated HIV-reactivation (**Fig. 7C and D**). In further experiments, a novel PKC inhibitor, rottlerin (inhibitor of PKC-δ, θ at IC_50_ of 3-6 µM) was able to ablate the bryostatin induced HIV-reactivation in a dose dependent manner, thus implicating PKC-δ and/or θ (**Fig. 7C, D**). Further, degradation of PKC is dependent on its kinase activity and activation of PKCs triggers its own degradation via the ubiquitin-proteasome pathway [52], [53]. Hence, we measured the total PKC levels using western blot in order to investigate whether bryostatin treatment could modulate degradation of these isoforms. Following treatment with bryostatin, time dependent depletion of total PKC-δ and PKC-α that is an indicator of PKC activation, was observed in THP-p89 cells (**Fig. 7E–G**) whereas other PKC isoforms were unchanged (data not shown). Hence, on the basis of PKC activation and inhibition studies, we concluded that novel PKC isoform, PKC-δ is involved in the reactivation of HIV.

### Bryostatin Mediated-HIV-Reactivation Involves Activation of PKCs via AMPK

**Figure 8:**
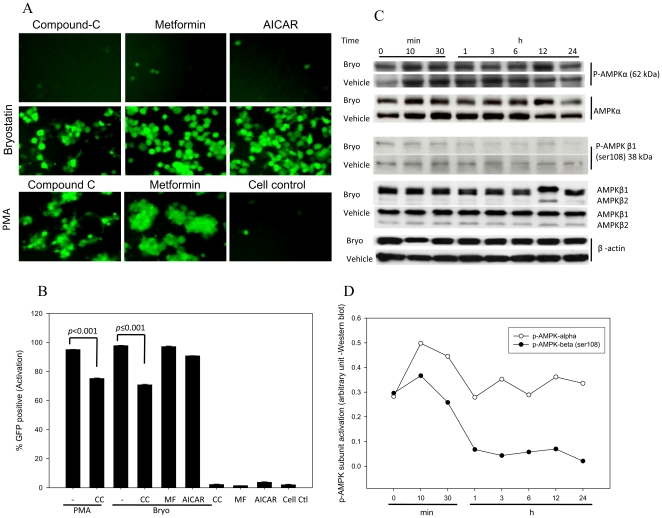
AMPK mediate reactivation of latent HIV infection via PKC. (**A**) GFP-expression in THP-p89 cells upon treatment with 10 µM Compound-C (CC), an AMPK inhibitor; 1 mM Metformin (MF) and AICAR (AMPK activators) either alone or in combination with bryostatin. Activation of AMPK alone does not show viral reactivation. (**B**) Quantification of THP-p89-reactivated GFP-positive cells by flowcytometry. (**C**) Western blots for phosphorylated AMPK-alpha subunit after treatment with bryostatin (25 ng/ml) were monitored at different time points. (**D**) Quantification of phospho-alpha and -beta subunit bands on western blots. Bryostatin treatment dephosphorylates AMPK-regulatory subunit β in a time dependent manner.

AMP-activated protein kinases (AMPK) are known to be involved in energy homeostasis by regulating the lipid biosynthetic pathway. AMPKs are activated by an elevated AMP/ATP ratio in response to various cellular and environmental stresses [54]. Antidiabetic drugs such as metformin and AICAR have been shown to upregulate AMPK, which play a significant role in PKC activation [55], [56]. In order to implicate the AMPK pathway in bryostatin mediated reactivation of latent HIV-infection, we used an AMP competitive inhibitor, compound-C that blocks the AMP binding γ subunit of AMPK. Compound-C treatment moderately ablated the bryostatin or PMA-mediated latent reactivation but not by metformin/AICAR (AMPK activators), suggesting the involvement of AMPK in part (**Fig. 8A, B**). AMPKs are highly conserved heterotrimeric protein complexes containing a catalytic α subunit and β/γ subunits involved in target and regulatory functions. We further investigated the activation and expression levels of both α and β subunits of AMPK in bryostatin treated THP-p89 cells. Bryostatin increased phosphorylation levels of the regulatory β1 subunit at serine-108 residue within 10 min of treatment (**Fig. 8C, 8D**). Furthermore, phosphorylation of this subunit was accompanied by phosphorylation at threonine 172 catalytic AMPK α subunit that was sustained at basal levels (**Fig. 8C, D**).

**Figure 9:**
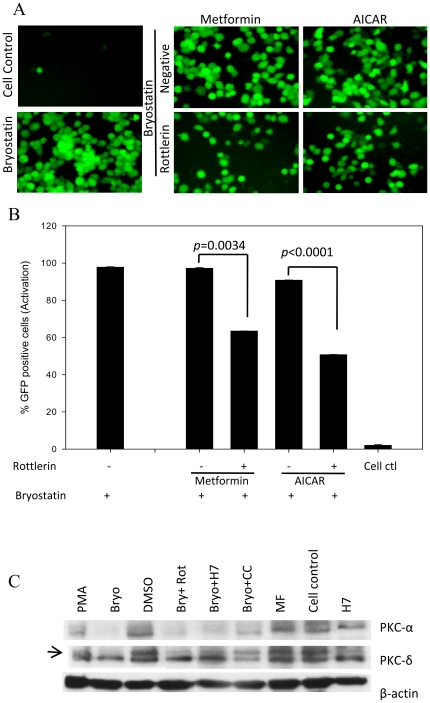
PKCs regulate reactivation of latent HIV infection downstream of AMPK. (**A** and **B**) nPKC (novel PKC) inhibitor, rottlerin abrogated viral reactivation in THP-p89 cells after AMPK activation (metformin and AICAR). Activation of AMPK alone did not affect viral reactivation. (**C**) Bryostatin- and PMA-mediated activation of PKC led to complete degradation of PKC-α and PKC-δ compared to vehicle control. H7 dihydrochloride and rottlerin (positive controls) mediated inhibition of activation of classical and novel PKCs prevented the degradation of total PKCs. Metformin an AMPK activator had no effect on the PKC, however, the inhibitor of AMPK, compound-C (inhibits AMPK activation) rescued the PKC degradation or prevented PKC activation in 24 h.

Remarkably, pretreatment of latent HIV-infected monocytic cells with nPKC inhibitor rottlerin followed by AMPK activators metformin or AICAR treatment, abrogated the bryostatin mediated HIV-reactivation (**Fig. 9A, B**). This infers that PKCs are the main effector kinases downstream of AMPK. Moreover, using Bryostatin or PMA as a positive control for PKC activation, there was a decrease in the phosphorylation levels of the beta subunit of AMPK compared to the vehicle control as shown earlier (**Fig. 8C and D**). The involvement of AMPK via PKC isoforms was investigated by protein profiling using Western blotting. Treatment of THP-p89 cells either with PMA or Bryostatin led to the activation of PKC-α and PKC-δ and were flagged for degradation compared to cell and vehicle controls (**Fig. 9C**). However, compound-C mediated inhibition of AMPK rescued the PKC degradation within 24 h. Although, induction of AMPK by metformin had no effect on the PKCs, it seems that the AMPK was already activated at basal activation state in these cells (**Fig. 9C**). Thus, activated AMPK (metformin or AICAR) regulates HIV reactivation in a PKC dependent manner.

### Bryostatin Synergizes Tat-Mediated LTR-Transactivation via Nef

**Figure 10:**
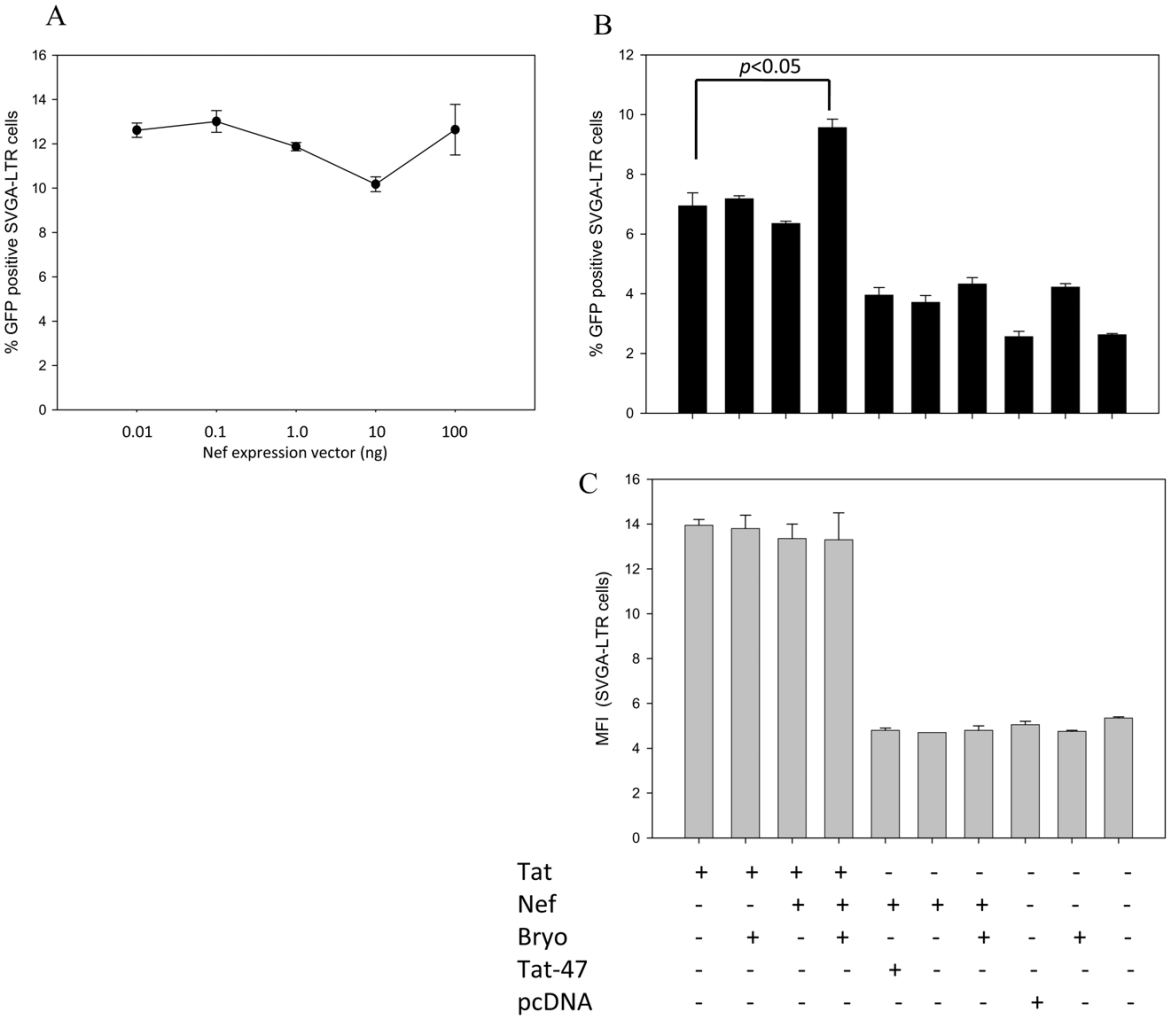
Bryostatin synergizes Tat- and Nef-mediated LTR-transactivation. SVGA-LTR-GFP reporter cells were transfected with 2 ng Tat-expression vector (suboptimal dose) and increasing concentrations of Nef-expression vector. (**A**) Quantification of GFP-positive cells. (**B**) SVGA-LTR GFP cells were transfected with 2 ng Tat-expression vector and 100 ng Nef-expression vector in the presence or absence of bryostatin (25 ng/ml) followed by GFP quantification using flowcytometry. (**C**) Mean fluorescence intensity (MFI) for the same experiment (**B**). (n = 3).

PKCs (novel) are known to mediate the subcellular localization of Nef and phosphorylation of Nef protein in the N-terminal region at arginine 6, resulting in activation of HIV-transcription using suboptimal concentrations of Tat [57], [58]. Hence, to have a proof of principle, we hypothesized that bryostatin could in fact facilitate HIV transcription via Nef protein. To validate this hypothesis, we performed co-transfection experiments using suboptimal concentrations of Tat expression vector (2 ng) with increasing concentrations of Nef expression construct (0 to 100 ng) on LTR-GFP reporter (SVGA-LTR-GFP) cells. Neither Nef nor bryostatin alone conferred any additional effect on Tat-mediated HIV LTR-transactivation (**Fig. 10A**). However, bryostatin treatment after co-transfection of wild type Tat but not mutant Tat (negative control) with Nef moderately potentiated LTR-transactivation in SVGA-LTR-GFP cells (**Fig. 10B**), suggesting that bryostatin could synergize with Nef and Tat in transcription. Although, there was a moderate increase in the number of GFP-positive cells by bryostatin and Nef, there was no increase in the mean fluorescence intensity (**Fig. 10C**).

### Bryostatin Induces PKC Activation without T-Cell Activation

**Figure 11:**
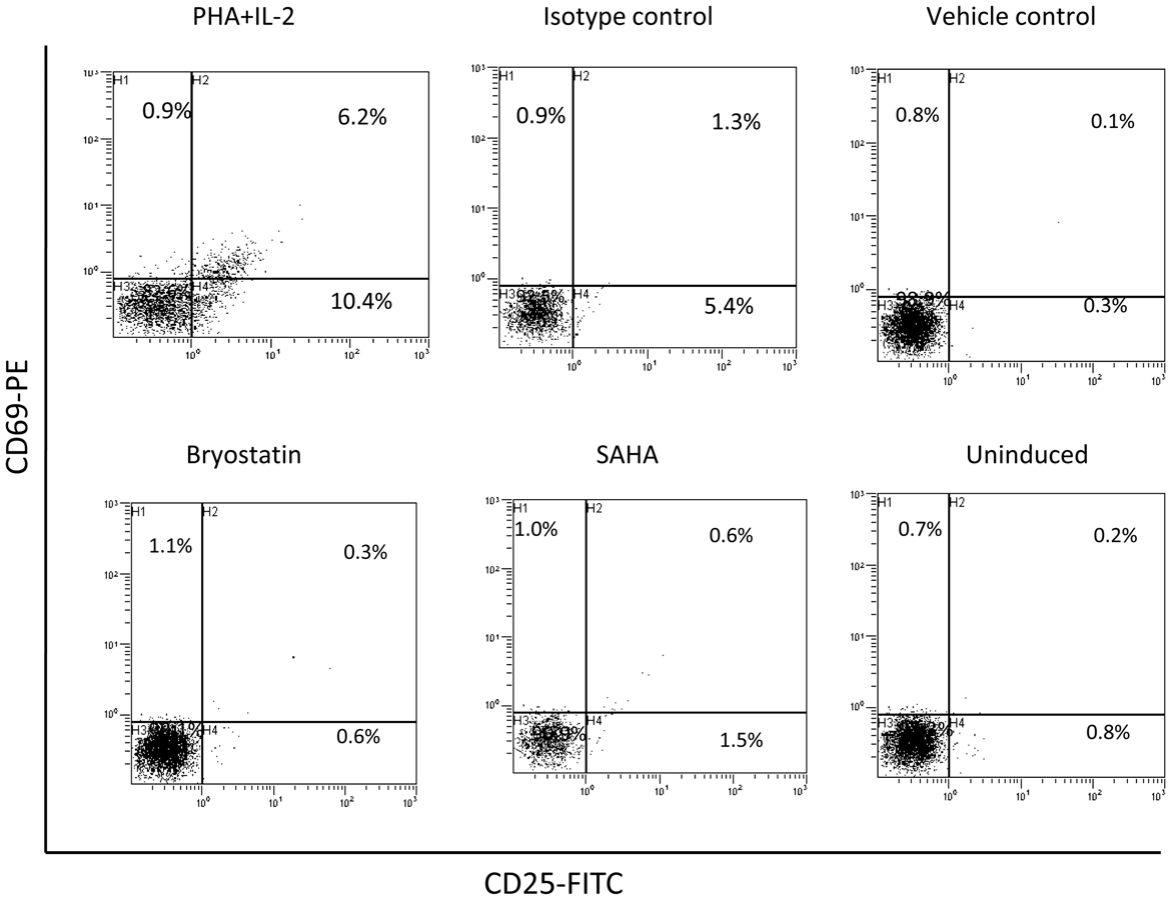
Effect of bryostatin on T-cell activation. Human PBMCs depleted of monocytes were cultured for 3 days and either activated with PHA and IL-2 or treatment with bryostatin for 2 days, followed by immunostaining with fluorescently labeled human anti-CD25 and anti-CD69 antibodies and monitored by flowcytometry. Representative dot plots for PHA and IL-2 activation, isotype control and bryostatin treatment are shown (n = 2).

Current therapeutic agents employed in viral reactivation require micro molar concentrations and have limited reactivation efficacy. In vivo latent viral reactivators have shown poor performance in clinical trials and even in combination with other activators were found to be ineffective in complete depletion of viral reservoirs [50]. Besides, the major disadvantage of these therapeutic agents in-vitro is their propensity to non-specifically activate bystander T-cells. We therefore, investigated the T-cell activation potential of bryostatin on primary human lymphocytes using CD25 and CD69 as activation markers. Human CD4-Tcells were cultured in T25 flasks. After three days in culture, they were treated with PHA and IL-2, and T-cell activation was monitored by flow cytometry using antibodies against CD25 and CD69. In parallel, we performed T-cell activation studies with traditional agents used in latent HIV-reactivation. Bryostatin at its active concentration (25 ng/ml) failed to activate T-cells compared to SAHA and prostratin. Lymphocytes stimulated with PHA and IL-2 were used as a positive control (**Fig. 11**). These observations demonstrate that bryostatin is a novel compound for HIV reactivation without inducing normal T-cell activation compared to SAHA and prostratin.

## Discussion

Despite the suppression of detectable plasma viremia in HIV infected patients; roughly one in a million CD4+ T cells will establish a latently infected long lived cell population [59]. Several broad spectrum therapeutic molecules are being investigated as potential candidates to eliminate latent HIV-reservoirs. Various strategies such as T-cell activation including IL-7, [16], [17], Amphotericin B [60], PKC activating phorbol ester derivatives such as prostratin and plant derived products like Jatrophane diterpene SJ23B [61], [62] and histone deacetylase (HDAC) inhibitors such as SAHA and Valproic acid [36], [37], [63] and 5HN (via oxidative stress and NF-κB independent pathway) [7] have been shown to reactivate latent HIV-1 infection. However, none of these strategies provided sufficient reactivation to be considered viable candidates for clinical purposes.

Our investigations on phorbol ester derivative bryostatin-1 revealed mild inhibitory effect on X4 and R5 acute HIV infections (**Fig. 2** **,** **3**). Similarly, X4-tropic anti-HIV activity was consistent with earlier study results [47]. The inhibition was in part, due to down-regulation of CD4 and CXCR4 in lymphocytes as well as receptor independent (**Fig. 4** **,** **5**). Although, receptors started reappearing between 6–24 h of treatment, sustained attenuation of viral replication in our observations suggested that bryostatin was conferring antiviral activity through some other mechanism as well (**Fig. 4**). Intriguingly, it is possible bryostatin may mediate this effect by regulating innate defense system in cells and require further investigations. Bryostatin induces PKCs which are in fact responsible for a wide range of activities including cellular proliferation and activation of NF-κB [7], [64]. Recently, a non tumorigenic phorbol ester, isolated from the Samoan medicinal plant, Homalanthus nutans, prostratin has been reported to activate NF-κB via novel PKC isoforms [49], thus providing a rationale to be included in a HIV therapeutic intervention regimen. We therefore, compared the activation potential of these two signal transducers and demonstrated that bryostatin was potently active in reactivation of latently HIV infected cells at much lower concentrations than prostratin (**Fig. 6A–F**). Recently, Lint and coworkers [50] reported that co-administration of prostratin with HDAC inhibitors such as sodium butyrate, apicidin; MS-275 and scriptaid would synergistically reactivate latent HIV infection, suggesting their therapeutic potential. However, prostratin in further studies was proved to be toxic at its effective concentration [49]. Interestingly, bryostatin at its effective concentrations failed to activate primary human lymphocytes (PBMCs) (**Fig. 11**) and was extremely potent in reactivation of latent HIV-1 compared to prostratin and SAHA (**Fig. 6A–F**).

Approaches for activating gene expression in latently infected cells rely on either stimuli that activate resting T cells or Tat [65]. In the current study, bryostatin in the absence of Tat neither showed a direct effect nor any synergy with Tat protein on HIV LTR (**Fig. 6G–H**), but revealed that PKC isoenzymes were involved in the bryostatin-mediated latent HIV reactivation (**Fig. 7A, B**). PKC isoenzymes are classified into three subfamilies as conventional or classical (α,β1, β2, γ), novel (δ, ε, η, θ) and atypical (ζ, λ) depending on the subcellular localization, biochemical properties and substrate specificity [66]. We attempted to decipher which of the isoenzymes are involved in the latent HIV reactivation process. Using GF109203X and rottlerin, inhibitors of classical and novel isoenzymes, we found that novel PKCs were intimately involved in latent HIV reactivation process (**Fig. 7**). Among nPKCs, PKC-θ has been exclusively found in T cells and its role in TCR signal transduction is well established [64] whereas PKC-δ is ubiquitously expressed, therefore, its role in viral activation was expected.

Phosphorylation of PKC leads to its activation, subsequent binding to signal lipids and translocation from cytosol to the membrane [67]. After activation, the levels decline upon translocation to endosomes followed by ubiquitination leading to proteasomal degradation thus producing a brief pulse of PKC activity in response to stimulus [52], [53]. Rapid degradation of diacylglycerol further shortens the duration of PKC activation. Similarly, in our study, treatment with bryostatin or PMA led to activation that resulted in reduced levels of PKC-α and PKC-δ (**Fig. 7**).

During HIV reactivation, target cells get stressed leading to loss of energy. AMPKs are known to act mainly as a central switch point in energy regulation; however, their role in response to cellular stress or infection has not yet been elucidated. Moreover, accumulated evidence has suggested that AMPKs not only regulate metabolism of fatty acids and glycogen but are also involved in other cellular processes such as modulating EF2 and TSC2/mTOR pathways [68] including NF-κB activation [69]. Our observations suggest that although AMPKs themselves were not directly involved in viral reactivation (via metformin or AICAR), however, activated AMPKs were found to coordinate the process via PKC. This was demonstrated upon pretreatment with AMPK inhibitor compound-C that resulted in attenuation of either bryostatin- or PMA-induced HIV-reactivation in monocytic cells (**Fig. 8**). In addition, metformin or AICAR activated AMPK coordination in bryostatin mediated latent HIV reactivation which was abolished by novel PKCs inhibitor Rottlerin (**Fig. 9**). These observations inferred that novel PKCs are the key regulatory molecules which are coordinated through AMPKs. Further, we also observed a small but significant synergistic transactivation effect of bryostatin along with Nef and suboptimal concentrations of Tat (**Fig. 10**). PKCs (novel) are known to phosphorylate the N-terminal of Nef protein which in turn will increase HIV transcription [57] and might be the phenomenon occurring upon bryostatin treatment in our experiments.

**Figure 12:**
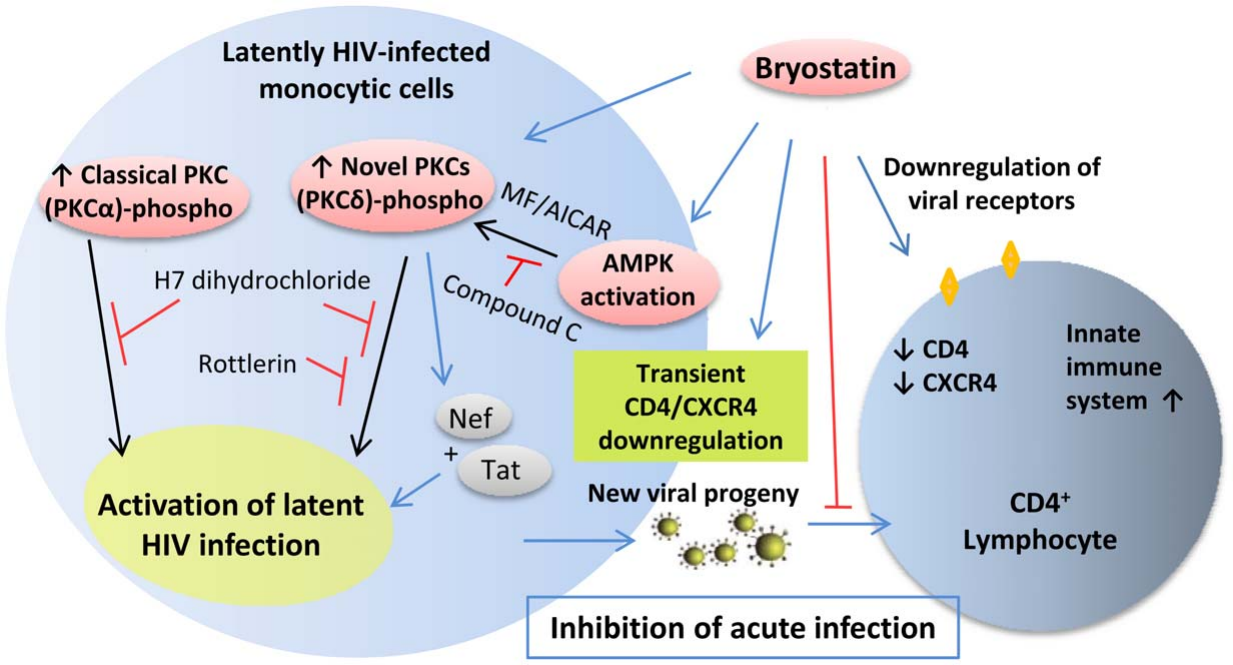
Schematic illustration of the mechanism of bryostatin action. Bryostatin treatment transiently down-regulate CD4 and CXCR4 receptors on lymphocytes/macrophages while intracellular activation of signal transduction pathways lead to reactivation of latent HIV infection. Bryostatin primed activation of classical and novel PKCs via AMPKs triggers reactivation of latent HIV-1. Involvement of activated innate immune defense system in curtailing HIV infection is expected.

The dual action of bryostatin inhibiting acute HIV-1 infection as well as reactivation of latent HIV via novel PKC pathway without T-cell activation and cytotoxicity makes it a promising novel therapeutic agent along with HAART to purge latent virus from tissue reservoirs (**Fig-12**). Future challenges will include the development and use of in-vivo systems aim at understanding the therapeutic behavior of bryostatin in combination with other drugs.

## Materials and Methods

### Chemicals and Reagents

Bryostatin-1 and prostratin were purchased from BioMol Research Laboratories, Inc. (PA, USA). SAHA was purchased from Cayman chemicals (MI, USA). GF109203X (Bisindolylmaleimide-1), rottlerin and H7 dihydrochloride were purchased from Calbiochem (CA, USA). Compound-C and 5-Aminoimidazole-4-carboxamide ribonucleoside (AICAR) were purchased from Toronto Research Chemicals, Inc. (ON, Canada). All other chemicals were purchased from Sigma Chemical Co. (MO, USA). The stock solutions of rottlerin (10 mM), H7 dihydrochloride and bryostatin (25 ng/ml) were prepared in dimethylsulfoxide (DMSO), and GF109203X (5 mM) was prepared in 50% DMSO in distilled water. AICAR (100 mM), metformin (100 mM), minocycline (10 mM), AZT (10 mM) and sodium salicylate (100 mM) were prepared in PBS followed by filter sterilization and aliquoted for one time use and stored at −20°C until used. Human anti-CD4, CXCR4, CD25, CD69 antibodies were purchased from BD-Biosciences (CA, USA). Anti-PKC isotype, phospho- and total-AMPK antibodies were purchased from Cell Signaling (MA, USA), anti-β actin antibody was purchased from Sigma, anti-rabbit-HRP and anti-mouse-HRP conjugate antibodies were purchased from Bio-Rad (CA, USA).

### Cell Culture and Latently HIV-Infected Cells

All cell culture (primary human and cell lines), HIV infection and proviral HIV DNA studies were performed according to university guidelines in a biocontainment facility approved by institutional biosafty committee (IBC) of the University of South Carolina. Peripheral blood mononuclear cells (PBMCs) were obtained from New York Blood Bank, Jurkat and J1.1 cells were from AIDS Research and Reference Reagent Program, NIH, and latently HIV-infected monocytic cell line THP-p89 [12] were maintained in RPMI (Gibco BRL life technologies, Inc., NY, USA) supplemented with 10% fetal bovine serum (FBS). HeLa, Magi (CD4/CCR5), TZM-bl cells (CD4/CXCR4/CCR5) were obtained through AIDS Research and Reference Reagent Program, NIH, and SVGA-LTR-GFP reporter cells [14], were maintained in DMEM containing 10% FBS. PBMCs were separated from normal human blood obtained through New York blood bank (NY, USA,) and cultured in RPMI-1640 containing 15% FBS. Monocytes were depleted by selective adherence to the plastic surface in culture plate and floating cells were cultured as lymphocytes [44]. Subsequently lymphocytes were stimulated with 1% PHA (Invitrogen Co., CA, USA) and 1 µg/ml IL-2 (R&D Systems, Inc., MN, USA).

### Cytotoxicity Assay

Cell Counting Kit-8 (CCK-8) (Dojindo Molecular technologies, Inc., MD, USA) was used in cell survival assays. CCK-8 utilizes Dojindo's highly water-soluble tetrazolium salt. WST-8 [2-(2-methoxy-4-nitrophenyl)-3- (4-nitrophenyl)-5-(2,4-disulfophenyl)-2H-tetrazolium, monosodium salt] produces a water-soluble formazan dye upon reduction in the presence of an electron carrier. WST-8 is reduced by dehydrogenases in cells to give a yellow colored product (formazan), which is soluble in the tissue culture medium. The amount of the formazan dye generated by the activity of dehydrogenases in cells is directly proportional to the number of living cells. Magi cells were seeded at a density of 2.5×10^4^ while Jurkat cells were plated at a density of 5.0×10^4^ per well in 96 well plate. On the subsequent day and thereafter, various chemical treatments were given in triplicate in a dose response manner. After 48 and 72 h, the cell supernatants were removed and 100 µl PBS and 10 µl CCK-8 reagent was added to the cells. After 3 h incubation at 37°C, supernatants were collected and the absorbance was measured at 450 nm wave length on multi-mode microplate reader (BioTek instruments, Inc., VT, USA). Percentage cell survival was calculated as OD_test_–OD_cell control_ and data were plotted as a mean of three tests and standard error of mean (SE) using sigma plot v8.0 (SPSS Inc.).

### Plasmid and Viral Constructs

HIV NL4-3 proviral-DNA wild type or envelope-deficient, p89.6 (dual tropic), NLENY1 and NLENYU2 carrying the YFP gene between the envelope and Nef in NL4-3 proviral DNA was used [12], [13], [45]. HIV wild type Tat (101 amino acids [aa]) and mutant-Tat 47 prepared by deleting 48–101 aa, were cloned at the BamH1 and EcoR1 sites in pcDNA vector. HIV-LTR was cloned upstream of GFP gene in CMV-promoter-deleted pEGFP vector and SVGA-LTR-GFP stable reporter cell line was generated [12], [14]. HIV NL4-3 and NLR^+^E^−^ proviral DNA, Nef (pcDNA3.1SF2-Nef) and VSV-G vectors were obtained through NIH AIDS Research and Reference Reagent Program, Division of AIDS, NIAID, Bethesda, USA.

### Viral Packaging and Pseudotyping

HIV NL4-3, HIV-NLENY1 and HIV-NLENYU2 and, NLR^+^E^−^ proviral DNA were used for packaging viral particles in 293T cells using HIV-proviral DNA alone or in combination with VSV-G expression vector [12]. Briefly, 293T cells were seeded in 100 mm tissue culture plates (BD Falcon, IL, USA). After overnight incubation cells were transfected with 17 µg of proviral DNA constructs either alone or in combination with 4.0 µg VSV-G expression vector, using Lipofectamine 2000 (Invitrogen). The supernatants were harvested 72 h post transfection followed by centrifugation at 300 g for 10 min and subsequent treatment with RNase free DNase-1 (Qiagen, CA, USA), and titrated for HIV p24 antigen (Zeptometrix Corp, Buffalo, NY, USA).

### HIV Infection

Testing anti-HIV activity of bryostatin and other compounds against R5-tropic strain, ∼1.5×10^5^ Magi cells (CD4^+^/CCR5^+^) were seeded in 24 well plates (BD Falcon™), while for X4-tropic HIV NL4-3, ∼2.5×10^5^ Jurkat cells were seeded in 24 well plate. The next day cells were pre-treated with various chemical reagents in optiMEM followed by infection with either NLENYU2 (R5) at 100 ng/ml equivalents- or with NLENY (X4) at 50 ng/ml equivalent -of p24 respectively for 2 h. The infected cells were washed twice and cultured for several days. The productive HIV infection was monitored semi-quantitatively by counting GFP-expressing cells in 10 random fields in each well as well as quantitatively by p24 ELISA. Appropriate controls were used such as Sodium Salicylate (1 mM) as a non-specific negative control, while AZT (100 µM) and minocycline (5 µg/ml) were used as positive controls. For testing receptor-independent HIV inhibition in single round infection, ∼3.5×10^5^ Hela cells (CD4 –ve) in 12-well plate were infected either with VSV-NLENY or VSV-NLR+E- HIV-1 prior to treatment with 25 nM bryostatin and appropriate controls. Testing HIV-infectivity after bryostatin treatment, supernatants were collected from HIV infected cultures treated with bryostatin and used for infection of TZM-bl (CD4^+^/CXCR4^+^) cells (∼3.5×10^5^) in 12-well plate. Virus production was monitored 3 day post infection by monitoring GFP fluorescence and viral p24 by ELISA.

### Flowcytometry

The quantification of reactivated latently HIV infected, acutely infected or Tat transfected LTR-GFP cells was performed after fixation with 3% paraformaldehyde for 30 min at 4°C followed by washing twice with PBS. The effects of bryostatin mediated re-programming of cell surface receptors on PBMCs or Jurkat cells were monitored by flowcytometry after staining with fluorescently labeled anti-human CD4, CD25, CD69, and CXCR4 antibodies respectively. Bryostatin or SAHA mediated T-cell activation (PBMCs) was monitored after staining for activation markers with fluorescently labeled anti-human CD25 and CD69 antibodies. Briefly, 1×10^6^ cells were washed with PBS and stained with 20 µl of fluorescently labeled antibodies in 100 µl PBS containing 1% FBS for 45 min on ice. Subsequently, cells were washed three times with PBS and finally resuspended in 1 ml PBS containing 1% FBS and 10,000 cells were acquired using FACScan flowcytometer with Expo32 v1.1B software (Beckman Coulter, Inc., FL, USA).

### LTR Transactivation and HIV Reactivation

SVGA-LTR-GFP reporter cells were established by stable transfection using HIV-LTR-GFP expression vector [14], monoclonal cells were cloned and used in transactivation studies. The reporter cells were seeded into 6 well plate followed by transfection with Tat expression vector in a dose responsive manner. A subsequent treatment with 25 ng/ml bryostatin was given followed by transfection with either Tat expression vector or mutant-Tat (defective in its transactivation function), which was used as a negative control. Each plasmid of 50 ng concentration was transfected using Lipofectamine 2000 (Invitrogen) as described earlier [14]. After 48 h of transfection, cells were stained with 1 µg/ml Hoechst-33342 for 15 min at 37°C and the fluorescence was monitored using a Nikon Eclipse fluorescence microscope. Results were quantified by either mean GFP florescence normalized with Hoechst fluorescence on BioTek synergy 2.0 microplate reader or by flowcytometry.

THP monocytic cells, which are latently infected with recombinant HIV p89-GFP [12], [15], [45], were seeded into 6 well plate at a density of 7×10^5^ cells per well. The basal viral activity in THP-p89 cells was barely detectable with complete absence of GFP-expression and p24. Viral reactivation was performed upon treatment either with TNF-α or bryostatin. After overnight cell seeding, cells were treated with TNF-α (positive control), bryostatin (25 ng/ml) or inhibitors of PKC isoforms at different concentrations either alone or in combination with bryostatin, in parallel with appropriate controls. After 24 h of treatment, cells were fixed with 3% paraformaldehyde followed by staining with 1 µg/ml of Hoechst 33342 dye for 15 min at 37°C and quantified by flow cytometry. The percentage of GFP expressing cells was used as a measure of viral activation/suppression. Each experiment was done in duplicate and a result of % GFP was expressed as a mean of two readings.

### Western Blotting

Latently HIV-infected THP-p89 cells were seeded in 6 well plates followed by treatment with 25 ng/ml bryostatin along with vehicle control (DMSO) and compound-C. After 48 h of treatment, cells were washed twice with ice cold phosphate buffered saline (pH 7.2) followed by centrifugation at 450 g for 5 min. Cell pellets were lysed with RIPA buffer (Sigma) supplemented with protease inhibitor cocktail (Pierce, IL, USA) on ice for 5 min. Subsequently, cell debris was removed by centrifugation at 12000 g for 2 min and the protein concentration was determined by BCA kit (Pierce). The supernatants (25 µg protein) were analyzed on SDS-PAGE and transferred onto PVDF membrane (Bio-Rad) followed by blocking with 5% milk (Bio-Rad) and 0.1% tween-20 in PBS buffer. The blotted membranes were incubated with appropriate antibodies against PKC or AMPK (phosphorylated or normal isoforms) and probed with anti-rabbit HRP conjugated secondary antibody (1∶10000). The blots were developed using ECL reagent (GE healthcare, PA, USA) and captured on to Kodak BioMax XAR film (Rochester, NY, USA). The protein bands were normalized to β-actin (1∶10000) and were quantified using ImageJ software (v 1.41; NIH).

### Statistical Analysis

Results are represented as mean ± SE for each bar graph using Sigma plot v8.0 with associated p values for each treatment group compared to their respective controls. Statistical analysis was done using origin 6.1 software. Significance between two groups was calculated using two tailed student's t-test followed by one-way analysis of variance. p<0.05 was considered significant.
